# Insulin and aging – a disappointing relationship

**DOI:** 10.3389/fendo.2023.1261298

**Published:** 2023-10-03

**Authors:** Hubert Kolb, Kerstin Kempf, Stephan Martin

**Affiliations:** ^1^ Faculty of Medicine, Heinrich-Heine-University Düsseldorf, Düsseldorf, Germany; ^2^ West-German Centre of Diabetes and Health, Düsseldorf Catholic Hospital Group, Düsseldorf, Germany

**Keywords:** insulin, insulin resistance, aging, longevity, senescence, oxidative stress, proteostasis, Nrf2

## Abstract

Experimental studies in animal models of aging such as nematodes, fruit flies or mice have observed that decreased levels of insulin or insulin signaling promotes longevity. In humans, hyperinsulinemia and concomitant insulin resistance are associated with an elevated risk of age-related diseases suggestive of a shortened healthspan. Age-related disorders include neurodegenerative diseases, hypertension, cardiovascular disease, and type 2 diabetes. High ambient insulin concentrations promote increased lipogenesis and fat storage, heightened protein synthesis and accumulation of non-functional polypeptides due to limited turnover capacity. Moreover, there is impaired autophagy activity, and less endothelial NO synthase activity. These changes are associated with mitochondrial dysfunction and oxidative stress. The cellular stress induced by anabolic activity of insulin initiates an adaptive response aiming at maintaining homeostasis, characterized by activation of the transcription factor Nrf2, of AMP activated kinase, and an unfolded protein response. This protective response is more potent in the long-lived human species than in short-lived models of aging research resulting in a stronger pro-aging impact of insulin in nematodes and fruit flies. In humans, resistance to insulin-induced cell stress decreases with age, because of an increase of insulin and insulin resistance levels but less Nrf2 activation. These detrimental changes might be contained by adopting a lifestyle that promotes low insulin/insulin resistance levels and enhances an adaptive response to cellular stress, as observed with dietary restriction or exercise.

## Introduction

Humans and most animal species exhibit the phenomenon of aging prior to dying a natural death. There is an age-dependent increase of physical damage to cellular constituents and changes in cellular and organ function.

At the cellular level, age associated damage includes the accumulation of defective macromolecules such as oxidized lipids, proteins and deoxyribonucleic acid (DNA), as well as the formation of protein aggregates. There is increased production of free radicals and less adenosine triphosphate (ATP) from dysfunctional mitochondria in the context of lower availability of nicotinamide adenine dinucleotide (NAD+) and altered nutrient sensing. Cell repair and turnover mechanisms are impaired as evident from impaired proteostasis, decreased autophagy and lower stem cell activity (1–4). In most cell types, cell division is accompanied by shortening of telomeres which may prohibit proper replication of chromosomes. Further, aging is associated with modifications of DNA and histones, and there is a strong correlation between methylation patterns of DNA and chronological or biological age (5). Several of these defects initiate cellular senescence, a functional state with replicative arrest, resistance to apoptosis, often associated with secretion of a variable combination of soluble factors and exosomes which promote low-grade inflammation, fibrosis and senescence of additional cells (4, 6). There is impairment of immune functions, termed immunosenescence. Probably all organs exhibit altered or deficient functions, including the microbiome (1–11).

Can a natural course of aging be defined? Is there a primary lesion which kicks off a cascade of defects, and what is the role of insulin in this process? First of all, there is a genetic basis to the duration of life, otherwise the strikingly different lifespans between species such as between mice and humans or frogs and turtles could not be explained (12, 13). However, follow-up studies of the aging process have as yet failed to identify a primary cause and a standard sequence of events leading to functional decline of cells, organs and the organism. It has been suggested that DNA damage is an early lesion preceding other defects such as increased levels of oxygen radicals (14). However, it cannot be excluded that intracellular free radicals contribute to the accumulation of damaged DNA. It could also be argued that the primary lesion is a defective DNA repair response which would also promote the accumulation of DNA lesions. Alternatively, an impaired ability to scavenge radicals might precede increased levels of oxygen radicals (15, 16). Because of the interdependence between DNA damage, mitochondrial dysfunction, increased levels of free radicals, deficient autophagy, telomere attrition, loss of proteostasis, enhanced pro-inflammatory gene expression and cell regenerative activities, these different processes probably are part of a functional network. Aging could then be viewed as deterioration of a physiological network active within and between cells rather than being due to one primary damage initiating a linear chain of molecular events (8).

In support of the network concept is the experience from anti-aging trials. The DrugAge database of aging-related drugs lists several hundred compounds for which significant extension of the lifespan in at least one model has been reported. Drug targets include many different cell functions ranging from glutathione metabolism to synaptic transmission which argues against a dominant role of defects in only one cellular compartment (17). Similarly, genes associated with increased longevity code for many different cellular functions rather than for one critical process. Aging-associated genes are more likely to participate in the crosstalk between different pathways or biological processes, and there seems to be a network of “aging genes” directly interacting with each other (18).

**Figure 1:**
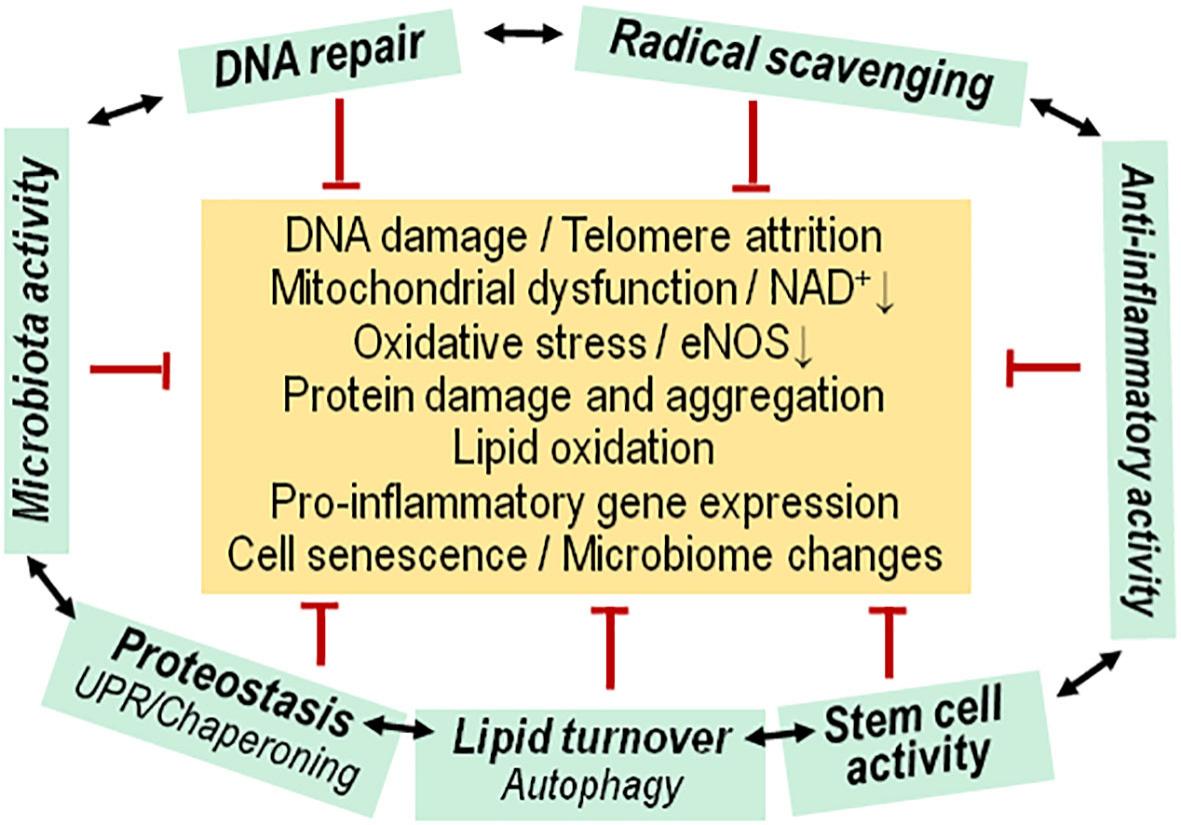
Functional network of cytoprotective pathways versus aging associated insults. Living cells experience a insults that usually initiate (green arrows) an adaptive, protective/repair response (red arrows) for maintaining cell functions, or there is replacement by newly differentiated cells. During the aging process, the adaptive response fails to maintain a normal physiological state of cells, Progenitor/stem cell activity is diminished, and there is concomitant dysfunction of the microbiota. Mitoch., mitochondrial.

We conclude that cell, organ and organismal physiology has several “weak spots” with low resistance towards metabolic, inflammatory, toxic or other types of stress. For instance, depending on genetic background, environment, lifestyle or developmental stage, there may be accumulation of DNA damage in excess of DNA repair capacity, protein aggregation during periods of high peptide synthesis overburdening protein turnover or disaggregation mechanism, high levels of oxygen radical formation in the context of intense mitochondrial activity and failing radical scavenging responses, or accumulation of oxidized lipids because of deficient autophagy (**Figure 1**). Such conditions may arise as consequence of high anabolic cell activity such as in response to excess concentrations of anabolic hormones like growth hormone, insulin-like growth factor (IGF) or insulin. The growth hormone – IGF – insulin signaling axis is a major modulator of the aging process (19, 20). In the present review we focus on the role of insulin which differs from that of growth hormone and the IGF system in that it is strongly linked to nutrient sensing. We suggest here that the age-associated decrease of resistance towards cellular stress may explain the unfavorable effects of insulin during aging. Thus, the actions of insulin may promote aging because of insufficient ability to cope with the cellular stress incurred by the hormone’s anabolic function.

## Insulin and aging: genetics

There is a strong genetic basis to an aging-promoting effect of insulin or the insulin/IGF-1 signaling pathway. A single mutation with impact on the insulin/IGF-1 signal transduction pathway, either affecting the sole insulin/insulin-like growth factor receptor or the phosphatidyl-inositol-3-OH kinase (PI3K), more than doubles the natural lifespan of the nematode *Caenorhabditis elegans* (21–23). Lifespan regulation by the insulin/IGF-1 signaling pathway is similar in the fruit fly *Drosophila melanogaster*. Genetic interference with proper signal transduction by various approaches shares as outcome an extension of lifespan (24).

In mice or humans, the regulation of the insulin/IGF-1 signal transduction pathway is more complex because of an additional upstream anabolic hormone, growth hormone. This pituitary hormone promotes IGF-1 production from the liver and other tissues, but the two hormones have partly opposite effects. For instance, growth hormone induces insulin resistance but promotes insulin production whereas IGF-1 promotes insulin sensitivity and reduces insulin secretion (25). Therefore, outcomes of genetic disturbance of the regulatory balance between growth hormone, IGF-1 and insulin are difficult to interpret. In mice, disruption of the insulin receptor in adipose tissue was sufficient to increase median and maximum lifespan by 18% (26). A body-wide knockout of the insulin receptor leads to early postnatal lethality whereas mice heterozygous for mutant and wildtype receptors did not show an altered lifespan despite some functional impairment of insulin signaling (27). In another study, mice heterozygous for a knockout of the insulin receptor showed no differences in lifespan to wildtype littermates in females but an increase in maximum lifespan in males (28).

Many studies have observed an extended lifespan in mice if growth hormone expression, or binding to its receptor are impaired. Longevity is increased in both sexes of Ames or other dwarf mice with deficient production of growth hormone together with prolactin and thyroid stimulating hormone or with isolated growth hormone deficiency (29, 30). Mice with disruption of the growth hormone receptor gene express a similar phenotype (31). The longevity mechanism of mice with deficient growth hormone activity has not been fully elucidated, but it is of interest that there is a strong association with enhanced insulin sensitivity (32). Similar analyses of IGF-1 are hampered by the fact that lack of functional IGF-1 receptors severely impairs development. Therefore, mice heterozygous for a receptor gene knockout were analyzed. Prolongation of lifespan was modest and seen in female mice only (33–36). IGF-1 receptor function can also be affected by deletion of insulin receptor substrate genes. This approach also impairs insulin signaling. Mice lacking insulin receptor substrate 1 exhibit increased longevity (37). For the insulin receptor substrate 2 gene, deletion in all tissues of mice was not found to increase lifespan while deletion in brain tissue only promoted longevity (38) (**Table 1**).

**Table 1:** Genetic manipulation of anabolic hormone signaling versus lifespan.

Organism	Effect	References
***Caenorhabditis elegans***	Genetic impairment of the insulin/IGF-1 signaling pathway extends lifespan.	(21–23)
***Drosophila melanogaster***	Genetic impairment of the insulin/IGF-1 signaling pathway extends lifespan	(24)
**Mouse strains**	Genetic impairment of growth hormone expression or signaling extends lifespan	(25, 32, 33, 39)
	Genetic impairment of IGF-1 receptor expression modestly extends lifespan in females	(33–36)
	Genetic impairment of insulin receptor expression has modest or no effect on lifespan extension	(26–28)

The opposing effects of growth hormone and IGF-1 on insulin sensitivity and production leads to the question whether insulin action itself is more closely related to longevity than the two other anabolic hormones. In mice, modulation of circulating insulin levels and insulin sensitivity often but not always were reported to affect the lifespan which supports a role of insulin actions in the aging process. In one study, mice with reduced insulin sensitivity because of impaired insulin receptor function exhibited an increased lifespan in males but not in females. Increased insulin sensitivity because of deficiency of protein tyrosine phosphatase 1B or overexpressed peroxisome proliferator activated receptor gamma coactivator-1α was associated with a shortened lifespan (28). Another strain of mice with impaired insulin receptor function also exhibited insulin resistance and hyperinsulinemia, but without an impact on lifespan (27). Modest lowering of circulating insulin levels by 25 – 34% but not of IGF-1 *via* knocking out the Ins1 gene and one of two Ins2 alleles in female mice appeared to increase maximum lifespan (p < 0.059) (40).

In humans the contribution of single genes coding for components of the insulin/IGF-1 signaling pathway to longevity appears to be low with the exception of *FOXO3A* (41, 42) and possibly *AKT1* (43, 44). However, the genetic association of single nucleotide polymorphisms with human longevity became significant when polymorphisms of 68 genes of the insulin/IGF-1 signaling pathway were analyzed together. The significance of the association was carried by alleles of nine genes, *AKT1, AKT3, FOXO4, IGF2, INS, PIK3CA, SGK, SGK2*, and *YWHAG* (45). This study did not observe the well documented association of *FOXO3A* with longevity, possibly because nonagenarians rather than centenarians were analyzed.

Taken together, a low activity state of the insulin/IGF-1 signaling pathway promotes longevity, effects are stronger in nematodes and fruit flies than in mice or humans, possibly due to the more complex regulatory network in mammals which has as additional player, growth hormone, which is not present invertebrates.

Another additional factor determining the outcome of insulin actions on longevity might be the overall metabolic rate. A high metabolic rate is associated with increased production of reactive oxygen species (ROS). For instance, small-breed domestic dogs exhibit a higher mass-specific metabolic and growth rate than large dogs, and therefore oxidative damage of lipids is seen. Nevertheless, small-breed dogs live significantly longer (46, 47). In mice, heavier body weight is associated with increased epigenetic aging and earlier death (48, 49). Similar findings have been reported for humans. In Southern Chinese adults, the basal metabolic rate was inversely correlated with all-cause mortality in males, but not in females (50). Within a local population, people of smaller size have a higher life expectancy, in different regions of the world (51). It may be concluded that within a species a higher growth rate is associated with shorter lifespan, but this is not explained by a higher metabolic rate.

## Insulin and aging: epidemiological findings

In humans, epidemiological studies suggest a pro-aging effect of insulin. Insulin resistance increases with aging, but centenarians usually preserve normal glucose tolerance, low levels of fasting insulin and higher insulin sensitivity, when compared with adults > 75 years of age (52–54). The higher longevity in shorter men is also associated with lower fasting insulin concentrations (55).

In adults with normal glucose tolerance, there is a parallel increase of fasting insulin levels and insulin resistance with aging, and this is associated with central obesity (56, 57). Hyperinsulinemia and insulin resistance are important risk factors for type 2 diabetes as well as hypertension and cardiovascular disease (58–60). Age-related disorders associated with insulin resistance also include neurodegenerative diseases such as Alzheimer’s or Parkinson’s disease (61, 62).

Another approach of studying the health impact of hyperinsulinemia is to determine the insulinemic potential of the diet as assessed by food frequency questionnaires evaluated by measuring circulating C-peptide concentrations. Analyses of the prospective Nurses’ Health Study and the Health Professionals Follow-up Study (total of about 2,800,000 person-years) showed that a higher insulinemic potential of diet was associated with increased risk of all-cause, cardiovascular and cancer mortality (63). Of note, these associations were independent of BMI.

### Insulin and aging: (patho)physiological aspects

Insulin is a potent anabolic hormone. Just doubling fasting insulin levels is enough for suppression of lipolysis by approximately 50% and promotion of lipogenesis in adipocytes while hepatic gluconeogenesis is not yet inhibited (reviewed in (64)). A Mendelian randomization analysis found that genetic variants which code for a higher insulin response to glucose challenge are strongly associated with increased BMI which is considered as proof of a causal relationship between increased insulin secretion and body weight gain (65). This fits with the observation that insulin therapy favors weight gain (66). Conversely, pharmacological lowering of circulating insulin concentrations in obese people by diazoxide caused greater weight loss than diet alone (67). Treatment of obese persons with the somatostatin analogue octreotide led to weight loss in conjunction with a decrease of insulin levels (68, 69). Lifestyle changes or other interventions known to improve risk factors of age-associated disease and cardiovascular mortality cause lower insulin levels, as reported for calorie-restricted diets, intermittent fasting or bariatric surgery (70–73). Vegetarian diets are also associated with lower insulin resistance and lower fasting insulin levels, even in comparison with matched lean controls, and appear to improve healthspan and possibly also lifespan (74, 75). Another lifestyle parameter associated with better healthspan is physical exercise, which causes lower fasting and post-challenge insulin levels as well as improved insulin sensitivity (76–78).

Although insulin is an essential hormone for growth and maintenance of complex organisms (79), the above findings suggest that elevated insulin levels promote age-associated diseases. One cellular response to permanently elevated insulin levels is partial downregulation of insulin signaling *via* the insulin receptor, causing the phenomenon of insulin resistance. This may involve decreased insulin receptor expression, but the major reason is impaired signal transduction because of diminished tyrosine autophosphorylation of the receptor, removal of bound phosphate residues by phosphatases and suboptimal downstream signaling along the insulin receptor substrate (IRS) – (PI3K) – protein kinase B (PKB/AKT) pathway (80–83). A higher amount of alternatively spliced type A insulin receptor lacking exon 11 also may contribute to insulin resistance by directing insulin signaling towards the mitogen activated kinase pathway which promotes cell proliferation and tumor development (84).

Signaling *via* the PI3K-AKT pathway is not only affected by modulation of insulin receptor function but also enzyme activities downstream. The diversity of proteins involved in the PI3K-AKT signaling pathway allows for varying outcomes of signaling, and this complexity is only partially resolved. It therefore is not surprising that “insulin resistance” does not mean full suppression of hormonal activity but only downregulation of some insulin functions such as induction of glucose transporter translocation to the cell membrane (85, 86). In addition to impaired glucose transport, insulin resistance suppresses the stimulatory effect of insulin on nitric oxide production from endothelial nitric oxide (NO) synthase because of deficient posttranslational modification of the enzyme *via* PI3K/AKT activity (87, 88). The resulting decreased arterial smooth muscle relaxation is aggravated by the non-suppressed insulin-dependent influx of calcium ions which enhances vascular contractility, resulting in upregulated vascular tone which increases the risk of vascular events (89, 90).

**Figure 2:**
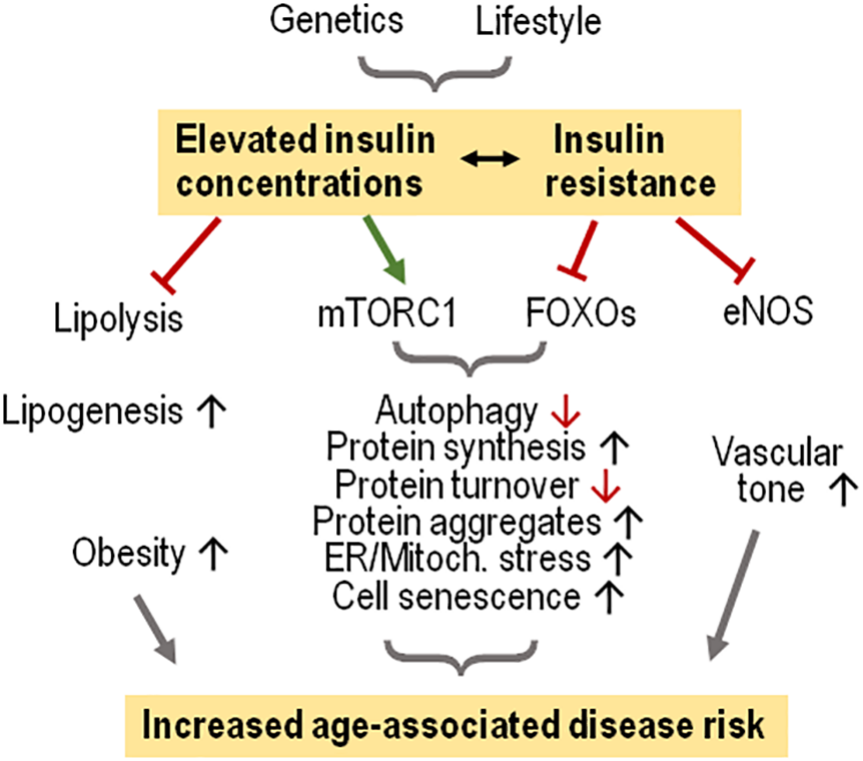
Elevated insulin levels and insulin resistance favor age-associated diseases in humans. Modest increases of insulin concentrations suffice to suppress lipolysis and support lipogenesis, promoting obesity. Hyperinsulinemia combined with insulin resistance cause activation of mTORC1 which in the context of less FOXO activation favors cell stress because of increased protein synthesis, eventually causing cell senescence. Insulin resistance impairs endothelial NO synthase (eNOS) activity, limiting vascular relaxation.

Other hormonal actions that are less or not affected by insulin resistance and may even be upregulated with the concomitant hyperinsulinemia include upregulation of PI3K-AKT dependent lipogenesis in hepatocytes and of the mechanistic target of rapamycin complex 1 (mTORC1) activity, the latter resulting in increased protein synthesis and impaired autophagy (91–95). Increased systemic insulin levels and concomitant insulin resistance during the progression to type 2 diabetes is associated with chronic overactivation of the mTORC1 signaling pathway and cell stress in the context of a high protein synthesis rate (96). During insulin resistance states (and concomitant hyperinsulinemia) there is, varying between tissues, phosphorylation of several Forkhead Box O (FOXO) transcription factors and their retention in the cytoplasm. resulting in suppression of muscle autophagy and protein degradation, among other effects (86, 97–99). The impact of elevated insulin levels on protein synthesis and autophagy is accompanied by the accumulation of proteins with multiple posttranslational modifications because of insufficient degradation which leads to endoplasmic reticulum stress (95, 100). Insulin signaling *via* phosphorylation of the Src homology 2 domain-containing transforming proteins (SHC) and subsequent activation of the mitogen-activated kinase protein kinase kinase (MEK) - extracellular signal-regulated kinase (ERK) is not affected by insulin resistance and contributes to these effects of hyperinsulinemia (**Figure 2**) (101, 102).

These findings suggest that increased insulin signaling because of elevated ambient levels causes cell stress, and there is a potentiating effect of insulin resistance. It therefore is not surprising that chronic exposure of human hepatocytes to high insulin levels (20 nmol/l) *in vitro* elicits a senescent cell phenotype, characterized by cell cycle arrest and adoption of a senescence-associated secretory phenotype which includes the secretion of proinflammatory mediators, microRNAs and vesicles (103). The promotion of hepatocyte senescence by hyperinsulinemia is absent in mice with a liver-specific knockout of the insulin receptor whereas enhanced senescence was still occurring in white adipose tissue. In obese persons undergoing bariatric surgery, insulin levels were closely associated with markers of senescence in liver tissue (104). Increased levels of insulin were also observed to promote senescence of human adipocytes *in vitro* as well as *in vivo* (105). High ambient insulin concentrations also drive mouse neurons into a senescence-like state, *in vitro* and *in vivo* (106).

Another age-associated marker is DNA damage. Prolonged incubation of animal or human cells with 0.5 nmol/l insulin caused DNA damage in the context of increased radical oxygen species production from nicotinamide adenine dinucleotide phosphate (NADPH) oxidase and mitochondria (107). Whether insulin resistance or the concomitant hyperinsulinemia promotes enhances telomere attrition in peripheral blood leukocytes in addition to cell stress has not been studied in detail. Cross-sectional studies suggest that that insulin resistance is associated with increased telomere shortening in some groups but not in others (108–112). A positive association was also noted in the follow-up of cohorts (113–115) with one exception (116). These observational studies also found an association between telomere attrition and other parameters such as adiposity, hypertension or circulating sirtuin-1 concentrations. Therefore, the association between telomere length and insulin levels may also be indirect.

## Insulin and aging: failure of adaptive response

As reviewed above, high insulin concentrations cause cell stress because of excess anabolic activity which include (i), increased lipogenesis and fat storage also in non-adipocytes, (ii), increased protein synthesis and accumulation of non-functional polypeptides because of limited turnover capacity, (iii), impaired autophagy activity, (iv) increased progression of stressed cells towards a senescent stage. These changes are associated with mitochondrial dysfunction and increased levels of radical oxygen species (117, 118). Hyperinsulinemia usually is accompanied by insulin resistance, but there is only partial suppression of insulin signaling, favoring lipogenesis as well as mTORC1 activation for protein synthesis and autophagy inhibition. The relevance of enhanced mTORC1 activation for the aging process has been demonstrated by treating mice with the mTORC1 inhibitor rapamycin which resulted in less proliferative and protein synthesis activity concomitant with improved autophagy and increased longevity. These changes resemble effects of dietary restriction. However, pharmacological inhibition of mTOR may reach a degree where detrimental consequences to the physiological balance are noted such as impaired immune cell activation, insulin resistance and beta islet cell damage (95, 119, 120). Insulin resistance in the presence of hyperinsulinemia helps maintain glucose homeostasis and decreasing metabolic and oxidative stress by depressing excess glucose influx (121, 122). However, the concomitant suppression of NO production from endothelial NO synthase favors a pro-oxidant and inflammatory vascular milieu as well as vasoconstriction potentially favoring vascular damage (**Figure 2**) (87–89).

**Figure 3:**
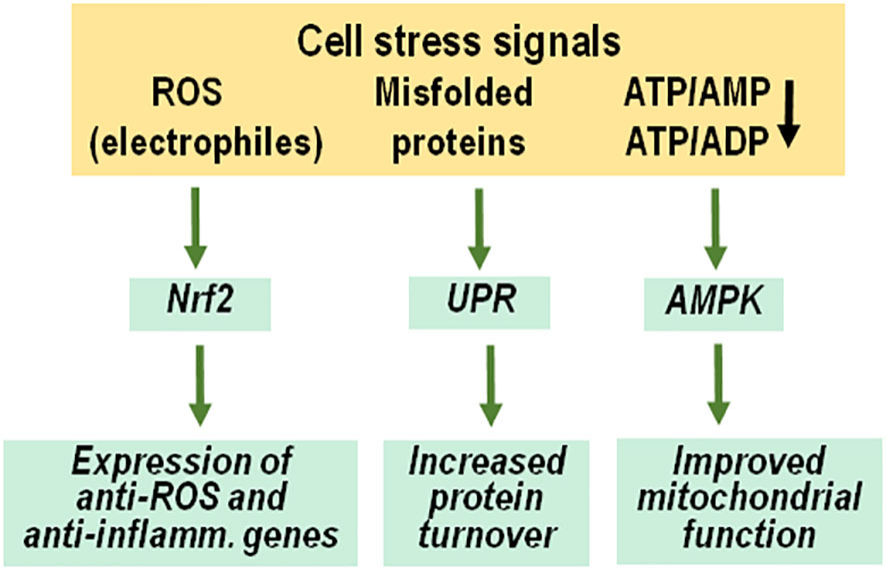
Adaptive response to anabolic cell stress. Molecular signals of cell stress include radical oxygen species and other electrophiles, misfolded proteins and decreased ATP/AMP and ATP/ADP ratios because of enhanced ATP consumption. These signals initiate an adaptive response to increase cellular resistance and restore proper physiological functions, including activation and nuclear transfer of Nrf2, an unfolded protein response and stimulation of AMP-activated protein kinases. ROS, radical oxygen species; UPR, unfolded protein response; AMPK, AMP-activated protein kinases, inflamm., inflammatory.

Taken together, hyperinsulinemia in the context of insulin resistance appears to exhibit a pro-aging role. Whether these effects become clinically relevant probably depends on the body’s ability to mount an appropriate defense response for containing the detrimental consequences of hyperinsulinemia and insulin resistance. One well documented health risk associated with increased insulin levels is type 2 diabetes. We have previously argued that the progression to overt type 2 diabetes is prevented if there is a persistent protective/adaptive response which includes an anti-inflammatory defense response to nutrient-induced inflammation, increased neutralization of free radicals and improved mitochondrial function for the reduction of oxidative stress, and an upregulated ability to lessen endoplasmic reticulum stress by an unfolded protein response and autophagy (123). We suggest here that this adaptive (hormetic) response also controls the pro-aging effect of insulin. The stress signals involved in inducing a hormetic response include oxygen radicals, misfolded proteins and decreased levels of ATP (**Figure 3**).

Oxygen radicals initiate a cell protective response by activation of nuclear factor erythroid 2 – related factor 2 (Nrf2), a key transcriptional factor for the expression of more than 250 genes involved in cytoprotective processes such as redox regulation, xenobiotic metabolism, DNA repair, and protein homeostasis including the unfolded protein response (124–126). There is impairment of pro-inflammatory gene expression, including the suppression of nuclear factor kappa B (NFkB) and pro-inflammatory cytokines (127, 128). Another effect of Nrf2 activation is the support of endothelial NO synthase expression and NO production (129). Thus, activation of Nrf2 is an appropriate adaptive cellular response to the oxidative, inflammatory and vascular stress caused by hyperinsulinemia and concomitant insulin resistance, with an impact on aging (130).

Loss of proteostasis because of excessive protein synthesis is a major consequence of an acute rise of insulin levels, but this is apparently contained by the unfolded protein response of the endoplasmic reticulum (100). Misfolded proteins signal the loss of proteostasis by binding to chaperone sensors which initiates a transcriptional program leading to a general increase of mechanisms involved in protein synthesis and turnover, the unfolded protein response (131). This protective cell response is impaired in the presence of experimentally induced or diabetes-associated insulin resistance (132). Low chaperone activity causes cell senescence (133).

A third important signal of cell stress is a decrease of ATP levels versus adenosine diphosphate (ADP) and adenosine monophosphate (AMP) concentrations, which results from increased consumption and deficient production of ATP. Low ATP levels lead to the activation of AMP-activated protein kinases. This group of kinases modulates the activity of many metabolic enzymes, histones and transcription factors by phosphorylation and by promoting their acetylation. One important consequence is the restoration of mitochondrial homeostasis (134–136).

As mentioned, several lifestyle factors have been observed to lower levels of fasting and postprandial insulin as well as of insulin resistance. These factors include dietary restriction and exercise (72, 137). Interestingly, dietary restriction or exercise cause an initial increase of oxidative or electrophile stress. The resulting activation of the Nrf2 system appears to mediate much of the health effects observed (138–141). Many dietary phytochemicals such as polyphenols also cause the activation of Nrf2, in part with an involvement of the hydrocarbon receptor (102, 142, 143). Another pathway of improving insulin resistance and concomitant hyperinsulinemia by lifestyle changes involves the gut, possibly by modulation of gut microbiota composition and activity may decrease gut leakage. The resulting lower levels of bacterial compounds in circulation is associated with decreased production of pro-inflammatory immune mediators and increased insulin sensitivity (144).

## Discussion

The anabolic hormone insulin induces cell stress because of increased biosynthetic activity and reduced clearance/repair of damaged cellular components. Insulin resistance is a potentiating factor because of increased signaling *via* the mitogen-activated kinase pathway and less production of NO by endothelial NO synthase. These potentially aging-promoting effects are contained by an adaptive cellular activity characterized by anti-oxidative, anti-inflammatory, protein chaperone, DNA repair and overall turnover process which is more potent in the long-lived human species than in short-lived models of aging research (145–147). Therefore, the pro-aging impact of insulin is less controlled in short-lived animal models such as nematodes and fruit flies. The balance between insulin/insulin resistance induced cell stress and the cytoprotective response determines detrimental effects of hyperinsulinemia and insulin resistance. Controlling factors are, on the one side, levels of insulin and insulin resistance, and, on the other side, the quality of cellular resistance to anabolic stress. This fits with the observation that centenarians exhibit low circulating insulin concentrations as well as high insulin sensitivity.

Of note, lifestyle factors that are considered to improve healthspan and possibly lifespan in humans modify both sides of the balance. Dietary restriction and exercise have been found to lower levels of insulin and insulin resistance. Concomitantly, dietary restriction, dietary phytochemicals and exercise activate the Nrf2-dependent cellular stress response and modify microbiota composition and function in a favorable way. During aging, the cell stress response *via* Nrf2 becomes less potent but possibly not in centenarians (148–151), and there is an age-dependent increase of circulating insulin and insulin resistance (52, 152). Both processes are supporting the pro-aging effects of insulin, and both may be targeted by dietary restriction and exercise.

## Author contributions

HK: Writing – original draft. KK: Writing – review & editing. SM: Writing – review & editing.
